# Surgical plate fixation of multiple rib fractures: a case report

**DOI:** 10.1186/s13256-018-1683-2

**Published:** 2018-05-29

**Authors:** Konstantin Mitev, Dashurie Neziri, Emil Stoicovski, Zan Mitrev

**Affiliations:** 1 Department of Orthopedic Surgery, Zan Mitrev Clinic, Skopje, Republic of Macedonia; 2 Faculty of Medical Sciences, University Goce Delchev, Shtip, Republic of Macedonia; 3 Department of Thoracic Surgery, Zan Mitrev Clinic, Skopje, Republic of Macedonia

**Keywords:** Multiple rib fractures, Surgical rib fixation, Reconstruction plates, Republic of Macedonia

## Abstract

**Background:**

The healthcare system in developing countries is limited; particularly, medical specialties such as emergency and trauma medicine are underdeveloped. Consequently, trauma injuries sustained in traffic accidents result in chronic morbidity more often than similar cases in developed countries. Multiple rib fractures induce significant patient morbidity. Current international guidelines recommend a multidisciplinary, surgery-based treatment approach to achieve optimal clinical benefit.

**Case presentation:**

We admitted a 41-year-old Albanian man to our emergency department following a pedestrian-vehicle accident 5 days earlier. He presented with severe upper thoracic pain, chest deformity, dyspnea, tachycardia, subcutaneous emphysema, and hematoma. Chest radiography pointed to hypoventilated lung fields and a minor pleural effusion. Computed tomographic scans indicated displaced fractures of right lateral ribs 5 –11, hyperdensity regions from bone fragments, and pulmonary contusion. The treatment consisted of surgical fixation of ribs 7–10 using titanium reconstruction plates and cortical locking screws. The patient’s clinical condition rapidly improved postoperatively. Follow-up at 6 weeks confirmed a full return to preoperative daily activities and a high quality of life.

**Conclusions:**

In this case report, we present a novel and promising development in the field of trauma medicine in the Republic of Macedonia. Trauma injuries can be treated via advanced multidisciplinary medical care according to international standards, allowing optimal health recovery.

## Background

The public healthcare system in developing countries struggles to follow international clinical guidelines because of inadequate medical infrastructure, lack of expert medical staff, and limited financial means. Emergency and trauma medicine are limited in developing countries such as the Republic of Macedonia [1, 2]. Consequently, trauma injuries remain a significant source of morbidity and mortality, in contrast to other Western countries [3]. Nevertheless, private healthcare practitioners support the public system and play a substantial role in the advancement of clinical practice in developing countries.

Blunt thoracic injuries sustained during road accidents frequently result in the fracture of one or more ribs. Rib fractures represent 20– 40% of trauma cases in emergency departments [4, 5], encouraging numerous researchers to assess the best practice guidelines. The recommended management of rib fractures has fluctuated over time, from conservative treatments based on external stabilization, analgesia, and respiratory support to internal surgical rib fixation. However, several lines of evidence indicate that surgical fixation complemented with a multidisciplinary bundled care pathway offers superior clinical benefit [6], particularly for patients who have sustained six or more rib fractures [4, 5, 7–9]. This case report describes an advanced multidisciplinary treatment provided by a team of anesthesiologists, trauma surgeons, intensivists, and physiotherapists that is unique to the Southeast Europe region. This case report signifies an imperative advancement in emergency and trauma medicine in the Republic of Macedonia.

## Case presentation

We admitted a 41-year-old Albanian man with dyspnea to our emergency department. He was complaining of severe upper thoracic chest pain after being involved in a pedestrian-vehicle traffic accident 5 days earlier. The patient was hit by a car while walking on the pavement, resulting in multiple rib fractures. Initial conservative treatments at another medical institution had failed to alleviate his symptoms. He had had recurrent episodes of fever in the days preceding his admission to our clinic.

The patient, a self-employed business owner, was still in emotional trauma upon his admission to our hospital. His medical history showed no evidence of narcotic addiction, but the patient’s family reported that he is a regular smoker and that he consumes above average amounts of alcohol.

Our examinations revealed an elevated blood pressure of 150/90 mmHg, mild tachycardia (95–105 beats/min), a chest deformity, subcutaneous emphysema, hematoma, and a score of 5 on a visual analogue scale (VAS) for pain [10]. From this point on, the patient’s pain intensity was assessed every 60 minutes; pain management was performed accordingly via intravenous analgesics because the patient was uncooperative with the epidural anesthesia.

Laboratory evaluations suggested possible liver trauma, indicated by elevated aspartate aminotransferase (AST) of 89 U/L and alanine aminotransferase (ALT) of 122 U/L, which was excluded by computed tomography. The patient had a normal renal function, Urea and Creatinine levels of 6.9 mmol/L and 64.4 μmol/L, respectively. The patient had normal body temperature (36.6 °C) and white blood cell counts (8.8*10^3^ cells/μl).

**Fig. 1:**
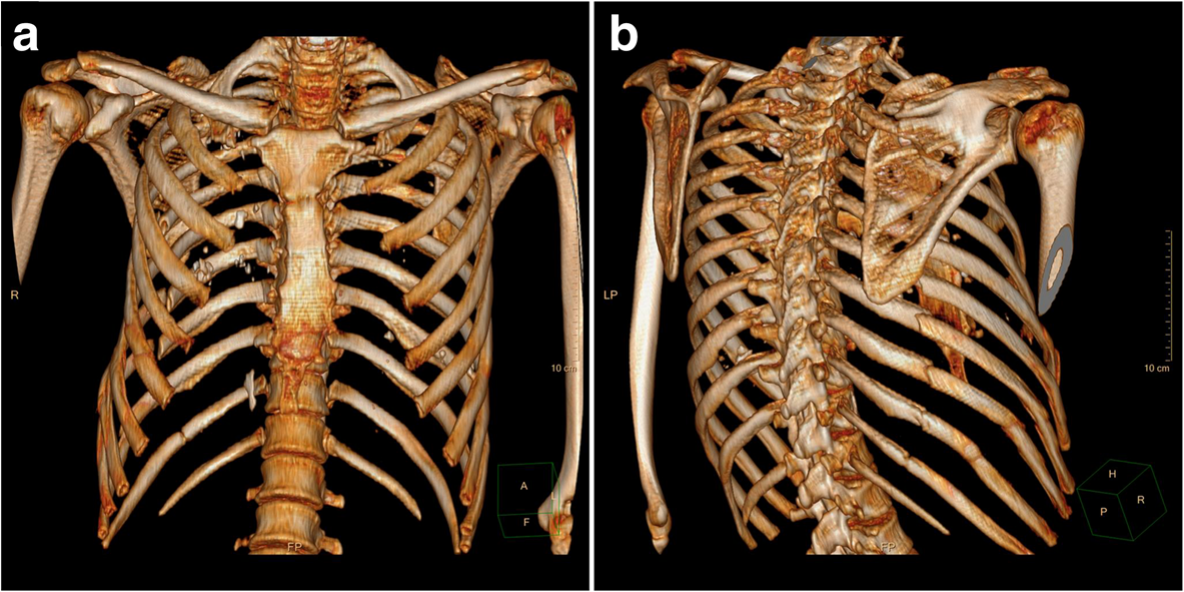
Preoperative three-dimensional reconstruction of the injuries from computed tomographic imaging. Three-dimensional chest computed tomography (**a**) anterior and (**b**) posterior reconstructions in a 41-year-old man who sustained fractures to ribs 5–11 on the right posterior side as a result of a traffic accident

**Fig. 2:**
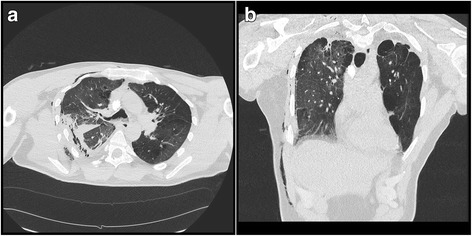
Preoperative computed tomography of the chest. Axial (**a**) and anterior (**b**) computed tomographic imaging indicating pulmonary contusion

We observed no neurological abnormalities. Chest radiography revealed possible fractures of several right lateral ribs, hypoventilated lung fields, and minor pleural effusion. Transthoracic echocardiography showed no other abnormalities. The patient’s left ventricular ejection fraction of 60% and blood pressure of 120/80 mmHg pointed to steady hemodynamics. Computed tomographic scans indicated displaced fractures of right lateral ribs 5–11 (Fig. 1), hyperdensity zones from bone fragments, and pulmonary contusion (Fig. 2).

We opted for general endotracheal anesthesia followed by an anterolateral thoracotomy. The transection was performed below the chest, above the costal margins, through cauterization of the pectoralis, serratus, and intercostal muscles. Four displaced ribs, the seventh through tenth, were stabilized using titanium reconstruction plates and cortical locking screws (small notch titanium reconstruction plates, thickness 3.5 mm, width 8 mm, catalogue number 489245; DePuy Synthes, West Chester, PA, USA). After thoracic drainage, 32 Fr, the surgery was terminated via standard closure of the intercostal wound incision and suturing overlying tissue. We transferred the patient to the intensive care unit following the surgery, where he stayed for 48 hours with no significant postoperative complications. Within the first 12 hours, the patient reported a low VAS pain score (< 2); as a result, we started with a rehabilitative physical therapy program from this point on. However, the patient experienced significant pain (VAS score of “6 – 8”) upon mobilization. He was more cooperative at this stage and allowed the placement of an epidural catheter between the fourth and fifth lumbar vertebrae for continuous infusion of bupivacaine (0.25%; 3–5 ml/h). The epidural analgesia decreased the patient’s VAS pain score to 2. Evaluation of the patient’s respiratory function confirmed a gradual recovery and full independence from the respiratory support system 48 hours after surgery (Table 1**)**.

**Table 1:** Intra- and postoperative respiratory parameters after surgical rib fixation following a blunt thoracic trauma

	Day 0	Day 1	Day 2	Day 3
Procedure	Surgery/patient on continuous mechanical ventilation support	Patient extubated	Arterial line removed	Initial mobilization
SpO_2_, %	97	94	96	95
Nasal oxygen support, L/min	FiO_2_ 45%^a^	4 to 6	0 to 4	0
Arterial saturation O_2_, %	95	93	90	ND^b^
PaO_2_, mmHg	98	78	63	ND^b^
PaCO_2_, mmHg	45	38	42	ND^b^
Respiratory rate, breaths/min	14	12	15	14

*Abbreviations: SpO*_*2*_ Peripheral capillary oxygen saturation, *FiO*_*2*_ Fraction of inspired oxygen, *PaO*_*2*_ Partial pressure of oxygen in arterial blood, *PaCO*_*2*_ Partial pressure of carbon dioxide in arterial blood

^a^Intraoperative oxygen support

^b^*ND* Not determined. Arterial saturation, PaO_2_, and PaCO_2_ could not be determined in the absence of an arterial line

**Fig. 3:**
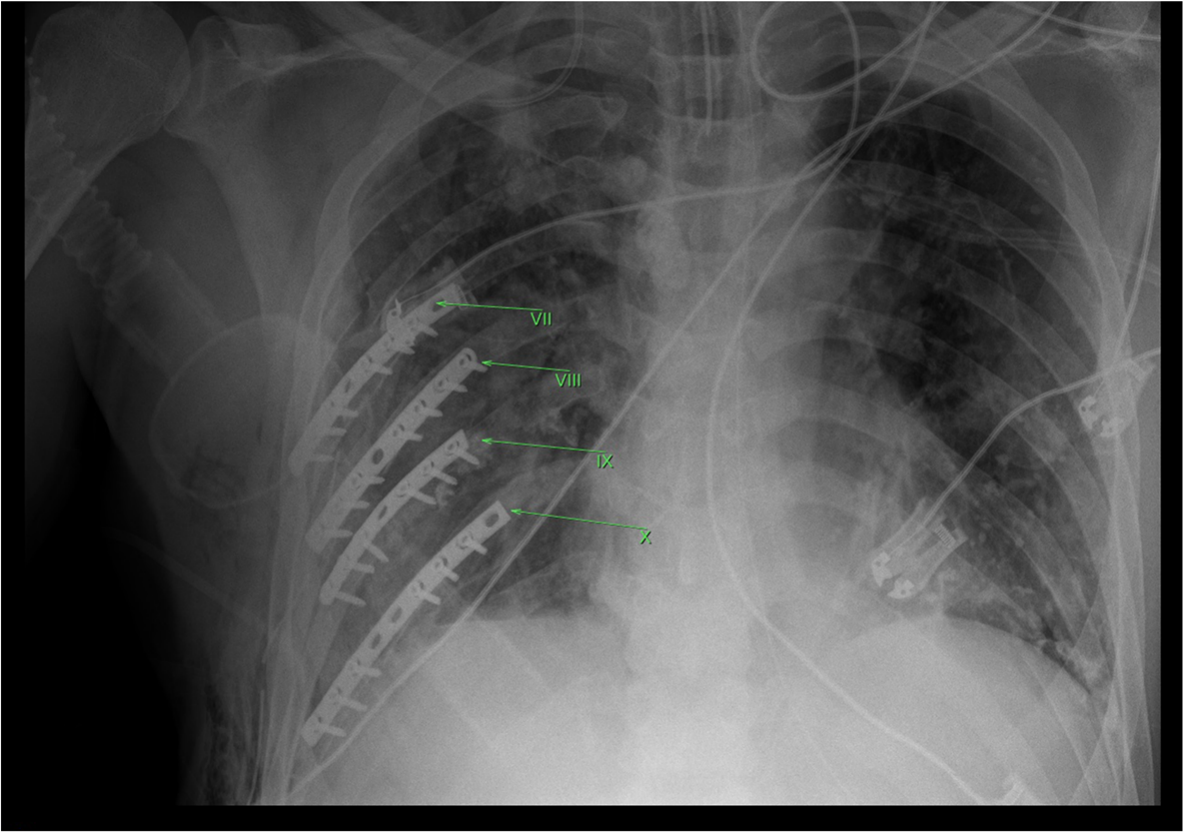
Postoperative chest x-ray of a patient with multiple rib fractures treated via thoracotomy and plate fixation. X-ray image displays the correct placement of the reconstruction plates and cortical locking screws on ribs 7–10

Control chest X-ray analysis on day 4 confirmed the correct placement of the plates (Fig. 3). Postoperative pain analysis on day 5 indicated a substantial relief from pain discomfort (VAS pain score of 1).

**Fig. 4:**
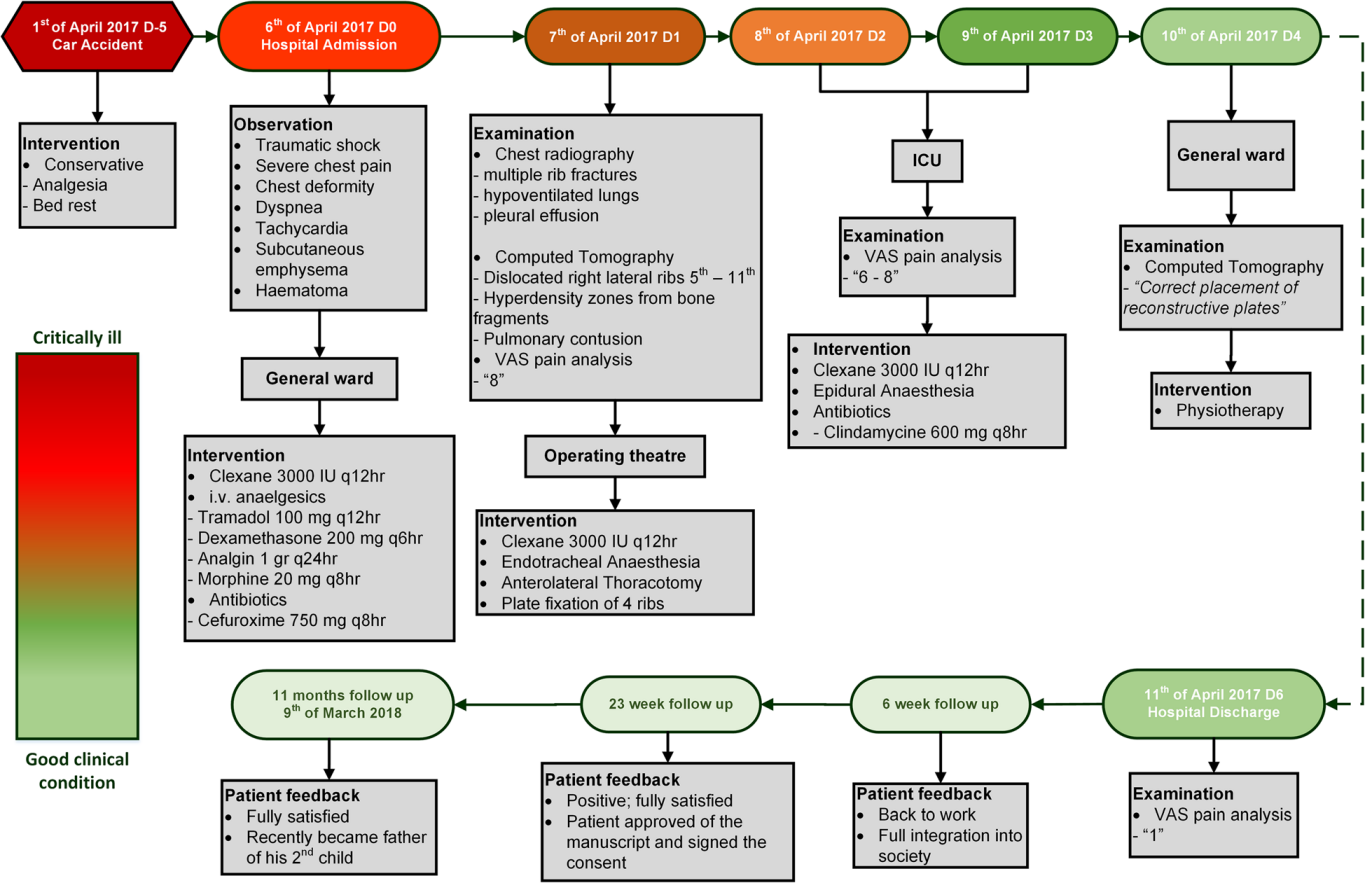
Hospitalization schedule, detailed treatment plan, and follow-up timeline. Bubbles at top show the specific dates on the timeline, and the colors indicate the clinical condition of the patient according to the color scale bar embedded in the figure. The bigger bubbles indicate key moments on the timeline. *Gray boxes* summarize the examinations; observations; interventions; medication dosage, schedule, and duration; treatment unit at the hospital; and patient feedback. *ICU* Intensive care unit, *VAS* Visual analogue scale

The total hospitalization was 6 days. Follow-up at 3, 6, 12, and 23 weeks and 11 months, via outpatient clinic visits and phone contact, confirmed a complete physical and mental recovery with no residual symptoms (Fig. 4). The patient reported that he was able to participate in work after 6 weeks and that he had quit smoking since the accident.

## Discussion and conclusions

This case report describes a novel and significant development in emergency medicine in the Republic of Macedonia. Multiple rib fractures resulting from blunt thoracic trauma are commonly treated through conservative means for socioeconomic reasons and because of limited health insurance coverage [1, 2]. To this end, the successful management of multiple rib fractures based on thoracotomy and surgical fixation is unique to our country.

Developing countries struggle to keep pace with the ever-evolving medical technology and practice guidelines [11, 12]. In the Republic of Macedonia, market reforms and public incentives have been in place to improve the efficiency and quality of primary health care since the fall of former Yugoslavia [13]. However, despite these measures, emergency and trauma medicine is poorly established [1]. Shared efforts and infrastructure across public and private sectors can help to promote advanced clinical practice and innovation in medical science.

As a consequence of the financial limitations and lack of resources, cases of multiple rib fractures are frequently treated conservatively in developing countries, despite the contraindications [5, 14, 15]. However, increasing numbers of multiple rib fractures correlate with pulmonary morbidity and mortality; patients who sustain fractures of six or more ribs are at significant risk for death resulting from causes (un)related to the rib fractures [16]. Therefore, the ideal clinical approach should be determined according to the severity of the rib fractures [4, 5, 7]. To this end, surgical fixation is currently the cornerstone of treatment for multiple rib fractures because it offers better postoperative pulmonary function, quicker verticalization and mobilization, and a higher quality of life [8, 9, 17, 18].

In light of the discussion above, this report presents a rare case of successful surgical fixation in the management of multiple rib fractures in a developed country that follows recent international recommendations and clinical practice guidelines [5]*.* Different methods [19] exist for surgical rib fixation, such as intramedullary nails [20], absorbable plating [21], Judet struts [22], Kirschner wires [23], titanium rib bridges [24], and plating with cortical screws [25]. There is no clear superiority of any of the methods. Nevertheless, the majority of reports describe the use of plating with cortical screws; our surgical approach adhered to the international trend. Indeed, follow-up examinations pointed to an effective surgical repair of four ribs using the reconstruction plates and successful patient recovery.

We treated the rib fractures using reconstruction plates instead of the precontoured locking plates of the MatrixRIB™ Fixation System (DePuy Synthes). As mentioned previously, in Macedonia, surgical plating of fractures is relatively rare, with few cases on an annual basis. To maintain cost-effectiveness and applicability to a broader patient population, we prefer the small notch titanium reconstruction plates because of their versatility. These plates can be applied to treat several fracture types, such as fractures of the radius, ulna, fibula, acetabulum, and metatarsals; certain cervical spine fractures; and, as described in this report, rib fractures.

A limitation of the treatment approach presented in this case report was the inability to complete the intended postoperative full-length rehabilitative physical therapy program because the patient resides at a considerable geographical distance from our clinic and has limited means of transportation. It is plausible that an intensive physical therapy regimen, individually tailored for patients recovering from surgery, could have sped up his recovery.

In conclusion, surgical plate fixation using small notch titanium reconstruction plates efficiently stabilized the patient’s ribs, mitigated his symptoms, rapidly improved his clinical condition, and promoted a swift reintegration into society. This report highlights a new and promising medical advancement in the Republic of Macedonia and Southeast Europe. Despite the stagnating progress of emergency medicine and limited national healthcare systems, trauma injuries can be treated successfully according to current international medical guidelines to provide the most optimal recovery possible.

## Abbreviation

VAS: Visual analogue scale
